# Investigation of Peptides for Molecular Recognition of C-Reactive Protein–Theoretical and Experimental Studies

**DOI:** 10.1021/acs.analchem.3c03127

**Published:** 2023-09-11

**Authors:** Katarzyna Szot-Karpińska, Patryk Kudła, Urszula Orzeł, Magdalena Narajczyk, Martin Jönsson-Niedziółka, Barbara Pałys, Sławomir Filipek, Andreas Ebner, Joanna Niedziółka-Jönsson

**Affiliations:** † Institute of Physical Chemistry, Polish Academy of Sciences, Kasprzaka 44/52, 01-224 Warsaw, Poland; ‡ Biological and Chemical Research Centre, University of Warsaw, Zwirki i Wigury 101, 02-089 Warsaw, Poland; § Faculty of Chemistry, University of Warsaw, Pasteura 1, 02-093 Warsaw, Poland; ∥ Department of Electron Microscopy, Faculty of Biology, University of Gdansk, Wita Stwosza 59, 80-308 Gdansk, Poland; ⊥ Institute of Biophysics, Johannes Kepler University, Gruberstrasse 40, 4020 Linz, Austria

## Abstract

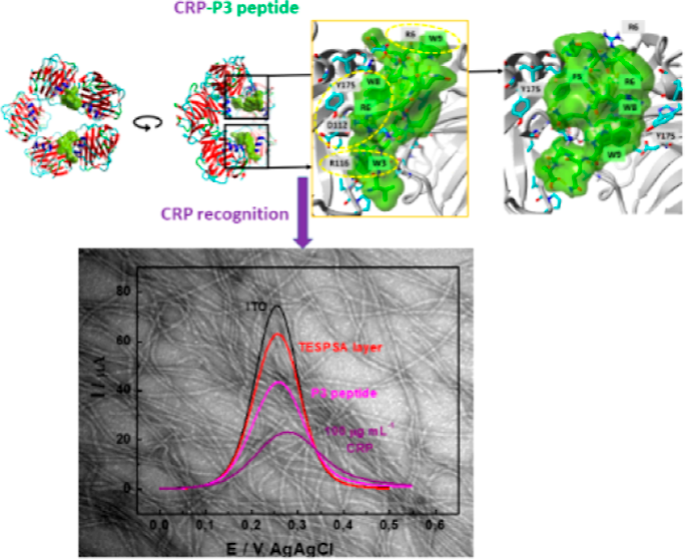

We investigate the interactions between C-reactive protein (CRP) and new CRP-binding peptide materials using experimental (biological and physicochemical) methods with the support of theoretical simulations (computational modeling analysis). Three specific CRP-binding peptides (P2, P3, and P9) derived from an M13 bacteriophage have been identified using phage-display technology. The binding efficiency of the peptides exposed on phages toward the CRP protein was demonstrated via biological methods. Fibers of the selected phages/peptides interact differently due to different compositions of amino acid sequences on the exposed peptides, which was confirmed by transmission electron microscopy. Numerical and experimental studies consistently showed that the P3 peptide is the best CRP binder. A combination of theoretical and experimental methods demonstrates that identifying the best binder can be performed simply, cheaply, and fast. Such an approach has not been reported previously for peptide screening and demonstrates a new trend in science where calculations can replace or support laborious experimental techniques. Finally, the best CRP binder—the P3 peptide—was used for CRP recognition on silicate-modified indium tin oxide-coated glass electrodes. The obtained electrodes exhibit a wide range of operation (1.0–100 μg mL^–1^) with a detection limit (LOD = 3σ/*S*) of 0.34 μg mL^–1^. Moreover, the dissociation constant *K*_d_ of 4.2 ± 0.144 μg mL^–1^ (35 ± 1.2 nM) was evaluated from the change in the current. The selectivity of the obtained electrode was demonstrated in the presence of three interfering proteins. These results prove that the presented P3 peptide is a potential candidate as a receptor for CRP, which can replace specific antibodies.

## Introduction

Over the past few years, peptides have gained ground in biomedical imaging,^1^ developing new sensing platforms^2^ and targeting treatments.^3^ This tremendous interest arises from their physicochemical and biological properties. Peptides are cheaper, obtainable in large amounts, easier to produce, more stable in harsh environments, more resistant to degradation than antibodies,^4^ and have comparable sensitivity.^5^ Moreover, antiviral, antifungal, antiparasitic, antitumor, and antibacterial properties are constantly discovered and evaluated.^6^ These peptides can be identified experimentally^7^ or designed through in silico analysis.^8–10^ Integrating these methods for developing peptide-based materials is an emerging trend in biomaterials science.^11^

As an experimental approach, the phage display technique allows for the identification of relevant peptides through the selection from a library of many phages exhibiting peptides that have an affinity to the particular target material, e.g., antigen.^11^ Once identified, such peptides can be produced quickly and cheaply by chemical synthesis.^5,12^ Peptides are relatively small molecules, and the possibility of further chemical modifications makes them useful in sensing as recognition elements. Short peptides have already been used for sensitive and selective assay of markers of various diseases, e.g., cardiovascular disease and ankylosing spondylitis.^12–15^ Peptides that bind specifically to proteins,^16^ viruses,^17^ and cancer cells^18^ have previously been identified using the phage-display technique. Banta et al.^13^ and Lee et al.^14^ indicate that the phage display technique makes it possible to identify phages with expressed specific peptides binding to markers associated with cardiovascular conditions. Then, such peptides are used to prepare sensing layers enabling sensitive, selective, and specific detection of these markers.^19,20^

In the fight against antibiotic resistance, improving the prevention, diagnosis, and treatment of bacterial infections is essential. Peptides that recognize CRP—a marker of inflammation in the human body—were a solution. CRP was discovered in 1930 by Tillett and Francis.^21^ It has a cyclic structure consisting of five identical, noncovalently connected subunits. Each subunit contains 206 amino acids. The molecular weight of the CRP protein is approximately 120 kDa.^22^ CRP is an acute-phase protein which is produced by inflammatory cytokines in liver cells, fat cells, and arterial walls,^23^ but an increased concentration of CRP in the blood is also observed in the case of cardiovascular diseases^24^ or cancer.^25^ There has been an increase in literature reports showing that CRP has the potential to be a marker of other diseases, e.g., rheumatoid arthritis,^26^ Alzheimer’s disease,^27^ or COVID-19.^28^ Therefore, there is a constant need to identify and characterize efficient receptors of this protein, which would be less costly and easier to obtain than the antibodies often used today.

Antibodies are currently the most frequently used CRP receptors.^24,29,30^ In the literature, there are also examples of CRP determination using bioreceptors such as aptamers,^31–35^ bacteriophages,^36^ affimers,^37^ peptides,^38,39^ or nanobodies.^40^

CRP assays are often performed using biological methods such as ELISA,^41^ test-based lateral flow immunoassays, and other immunoassays.^42^ These methods are costly and laborious and require expensive equipment and specialized staff. Additionally, sometimes the results are not clear-cut. Therefore, the analysis should be supported by other methods. A solution might be characterization of the molecular interactions of CRP with receptors using various physicochemical techniques. Some examples are 2D DNA origami nanostructures for quantitative single-molecule biosensing,^31^ SERS,^43^ fluorescence microscopy,^37^ atomic force microscopy,^43^ biolayer interferometry,^40^ or SPR^29,30,44^ and electrochemical methods.^36,40,45–47^ Not all of these are easy to perform,^31^ but they can give deeper analysis and insights into understanding protein–peptide interactions.

Our study used experimental (biological and physicochemical) and theoretical (computational modeling analysis) methods to study the molecular interactions between several new peptides and CRP. Only a couple of such peptides have been identified previously, one longer peptide (15 mer)^39^ and one very short peptide (3–5 mer) showing a very limited operation range.^48^ The 12 mer peptides binding to CRP were identified using the phage display.^38^ However, these peptides were combined with the two extra gold binding sequences (28 mer), so the final size of the peptide recognition element was 40 mer. Such a long peptide would not resist protease activity, limiting its application in vivo. In contrast, in our studies, for the first time, we demonstrated that a single 12 mer peptide panned from a phage library has been used for CRP recognition. The identified peptides were characterized, and their interactions with CRP were studied for application in a sensing platform.

The excellent correlation we show here between experimental and numerical methods is a powerful demonstration that the latter method can be used to simply, less costly, and quickly identify the best binder for a specific target. In our work, the computational analysis was not set to predict the peptide’s amino acid sequence because the sequences of selected peptides are known. However, the numerical analysis explains the details of the binding of selected peptides to CRP. Thus, the results reveal which peptide from a list of candidates is the best binder for CRP and confirm the experimental analysis. Therefore, integrating experimental studies with in silico studies can shorten the screening step, which may boost the research.

The integration of numerical methods with experimental studies is an emerging trend in science. It represents a significant improvement in developing peptide-based receptors and underlines the importance of simulations to support many laborious experimental techniques. Finally, the P3 peptide, which showed the highest affinity among the studied peptides, was used in an electrochemical sensor to illustrate its ability as a CRP recognition element.

## Experimental Section

### Electrochemical Analysis

Indium tin oxide-coated glass (ITO) electrodes [resistivity 8–12 Ω cm^–1^ (Delta Technologies)] were cleaned by sonication in a mixture of water and ethanol (1:1) for 20 min. The clean electrodes were functionalized using triethoxysilylpropyl succinic anhydride (TESPSA) for 4 h under an infrared lamp and heated in an oven (*T* = 120 °C) for 90 min. The silanization process was to create succinic anhydride groups that react with amines, thereby binding peptides via peptide bonds {1 h incubation of peptide [5.0 μg mL^–1^ in phosphate-buffered saline (PBS; 137 mM NaCl, 2.7 mM KCl, and 10 mM phosphate buffer, pH7.4)] or antibodies (2.5 μg mL^–1^ in PBS). This approach has already been applied to immobilize various biological components.^49^ The last step of the modification was blocking the remaining sites with 1% bovine serum albumin (BSA) in PBS by immersing the electrodes for 20 min at room temperature. The electrode surface was limited using Scotch tape (electrode area 0.2 cm^2^). The obtained electrode was incubated with 20 μL of studied protein [CRP, fibrinogen (Fib), troponin T (TnT), or interleukin 6 (IL-6)] dissolved in PBS for 30 min at room temperature to study molecular interactions between the immobilized peptide and studied proteins. Electrodes were rinsed with PBS (a few seconds) after each step. The amino acid sequences of the peptides are as follows; P2 (GGSDPEGMQGNY), P3 (VHWDFRQWWQPS), and P9 (SWFSDWDLELHA).

The electrochemical studies were performed in a three-electrode setup comprising a modified ITO working electrode, platinum wire counter electrode, and Ag|AgCl|KCl_sat_ as a reference electrode. A μAutolabIII (Metrohm Autolab) potentiostat powered by GPES 4.9 software was used for recording electrochemical data. Detection of CRP is based on the blocking of the electrode as the CRP binds to the immobilized recognition element [peptide or antibody (mAb)]. Measurements were performed with 1 mM ferrocenodimethanol (Fc(CH_2_OH)_2_) in PBS as a redox molecule using differential pulse voltammetry (DPV) (DPV parameters: scan rate 20 mV/s, step 4 mV, pulse amplitude 50 mV, pulse width 50 ms, and pulse interval 100 ms). The results are average values from three repetitions of the measurement at three separate electrodes, with RSD represented by error bars.

### Identification and Characterization of CRP-Binding Phages/Peptides

Details on identification of CRP-binding phages/peptides and characterization of interactions between CRP and new CRP-binding peptide materials using experimental methods are provided in the Supporting Information.

### Molecular Docking

The molecular structure of the CRP pentamer was taken from the Protein Data Bank id: 3PVO.^50^ The crystal structure of CRP was determined with a resolution of 3.0 Å, and all parts of the structure were visible in the crystal. The calcium ions, coordinated mostly by glutamic and aspartic acids in each subunit of the CRP, were removed.

The peptide–protein docking was performed in the ICM-Pro v.3.8 program (ICM-Pro; Version 3.8; Molsoft, L.L.C.: San Diego, CA, USA, 2020). As there is no experimental knowledge about peptide interactions with CRP complex, binding pocket searching was used.^51^ The pockets proposed by the program were ranked by volume as the interacting peptides are quite long. Four of the best-ranked pockets were used for docking.

The ICM docking procedure is based on the stochastic global optimization algorithm.^52^ The binding energy function was calculated from five grid potentials describing interactions of the flexible ligand with the receptor and the internal conformational energy of the ligand. The involved energy terms are van der Waals interactions for hydrogen atoms, van der Waals interactions for heavy atoms, electrostatic interactions, hydrophobic contacts, and the lone-pair-based potential, describing directional preferences in hydrogen bonds. The energy terms are based on the all-atom vacuum force field ECEPP/3 with appended terms to account for the solvation free energy and entropic contribution. The biased probability Monte Carlo (BPMC) procedure was used for the conformational space sampling of fully flexible ligands and side chains of the protein. The ICM program relies on global optimization of the entire flexible ligand in the receptor field and combines large-scale random moves of several types with gradient local minimization and a search history mechanism.

## Results and Discussion

Similar to our previous study,^36^ the phage-display technique was used to identify CRP binders. The affinity of the selected clones toward CRP was evaluated using two methods: plaque test (Figure S1A) and ELISA (Figure S1B).

The P3 clone resulted in the highest binding efficiency to CRP (2.17 × 10^–4^) based on the plaque test (O/I), expressed as the ratio of the eluted to added phages. This value is similar to that of the P2 phage obtained previously,^36^ also presented in this study. Their affinity is about 2 orders of magnitude higher compared to the wild-type phage (WT). The other clones measured, P1, P4, P6, P7, and P9, show low affinity to CRP, comparable with the WT phage (3.57 × 10^–6^, 5.00 × 10^–7^, 2.61 × 10^–7^, 1.33 × 10^–7^, and 3.13 × 10^–4^, respectively). P5, P8, and P10 are the weakest binders (7.50 × 10^–8^, 5.00 × 10 ^–8^, and 5.00 × 10^–8^). Their binding affinity is 4 orders of magnitude lower than that obtained for the P2 and P3 clones (Figure S1A).

In order to verify the affinity binding of the selected phages, an ELISA was performed. In this assay, the concentration of phages was determined by the enzymatic reaction of tagged anti-M13 antibodies. Analysis of CRP binding efficiency by measuring absorbance at 450 nm of selected clones (1–10) compared to binding for the WT phage using ELISA was performed and presented in Figure S1B. The selected clones have a higher affinity for CRP than for the WT (Figure S1B). The highest signal, 16, 14, and 13 times higher than for WT, was recorded for the clones P9, P6, and P3, respectively. Surprisingly, the P2 clones, which proved to be a good binder for CRP in the plaque test,^36^ exhibited a low affinity toward CRP in this test.

Based on both affinity binding tests, the P2, P3, and P9 clones were selected for further studies, and their exposed peptides were synthesized.

### TEM Analysis

Based on our previous studies, we know that M13 phage fibers—depending on the amino acid content of the exposed peptide—can form unique structures.^36^ Therefore, to find out what structures constitute the selected CRP-binding phage clones and peptides (P2, P3, and P9), TEM was used to visualize them. As depicted in Figure S2, the studied clones have the same dimension, and are similar to the length of wild-type M13, agreeing with the literature data.^36^ They form self-assembled, well-organized aggregates, and structures. However, the fibers of the selected phages formed by the association of the generated peptides interact differently. From TEM images, it can be seen that the fibers of the P2 and P9 are parallel to each other (Figure S2A,B) with the P2 fibers slightly intersecting. The P2 and P9 have predominant directions like anisotropic structures. In the case of P9, the observed structure is characteristic of a nematic phase in liquid crystals. The behavior of the filamentous M13 phages as liquid crystals is known in the literature.^53^ In the case of the P3 phage, apart from being adjacent, the fibers strongly intersect compared to P2, forming a more isotropic structure. Moreover, P3 forms a dense, cohesive spiderweb-like structure. In turn, P9 and P2 arrange in branchlike and loosely packed structures, respectively. These structures are different from those obtained for WT M13.^36^

Different structures result from the peptides exposed on the P2, P3, and P9 phage surfaces. The sequences of the modified peptides have various amino acids with different pI (3.8, 7.2, and 3.5—the pI was calculated using the free available software http://isoelectric.org/), respectively. So the physicochemical properties of these entities impact the interaction between phage fibers. The presence of aromatic amino acids like tryptophan, histidine, and tyrosine leads to additional stacking and van der Waals and/or hydrophobic interactions between phage fibers. In the case of the P3 peptide, these interactions prevail. The electrostatic interactions occur because of aspartic and glutamic acids, which are negatively charged, and histidine and arginine, which are positively charged. These interactions are predominant for P3 and P9, whereas for P2, these interactions are negligible.

**Figure 1:**
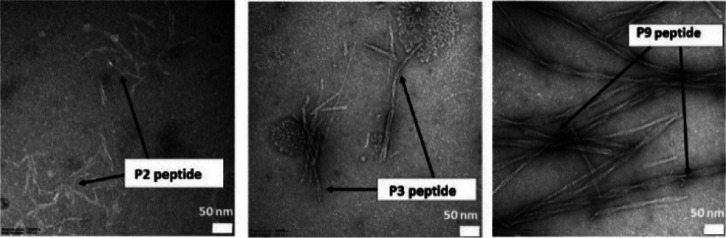
TEM images of fibers of selected peptides: P2 peptide, P3 peptide, and P9 peptide. Scale bar 50 nm.

Moreover, TEM images of bare peptide fibers were taken (Figure 1). As can be seen, the P2 peptide creates loose fibers, which are unordered and without forming a specific structure (Figure 1).

In the case of the P3 peptide, the structure is ordered. There, the fibers are self-assembled and generally set parallel to each other (Figure 1). In contrast, the structure and the morphology of the P9 peptide fibers differ significantly from the P2 and P3 peptides. In the P9 peptide fibers, contractions are visible due to changes in orientation. The fibers self-assemble into unique structures called twisted nanoribbons. The formation of such structures is driven by β-sheet hydrogen bonds typical for peptides with β-sheet structures.^54^ Such a structure has been confirmed only for the P9 peptide in this study using PM-IRRAS analysis (Figure 3C).

### Molecular Docking Analysis

**Figure 2:**
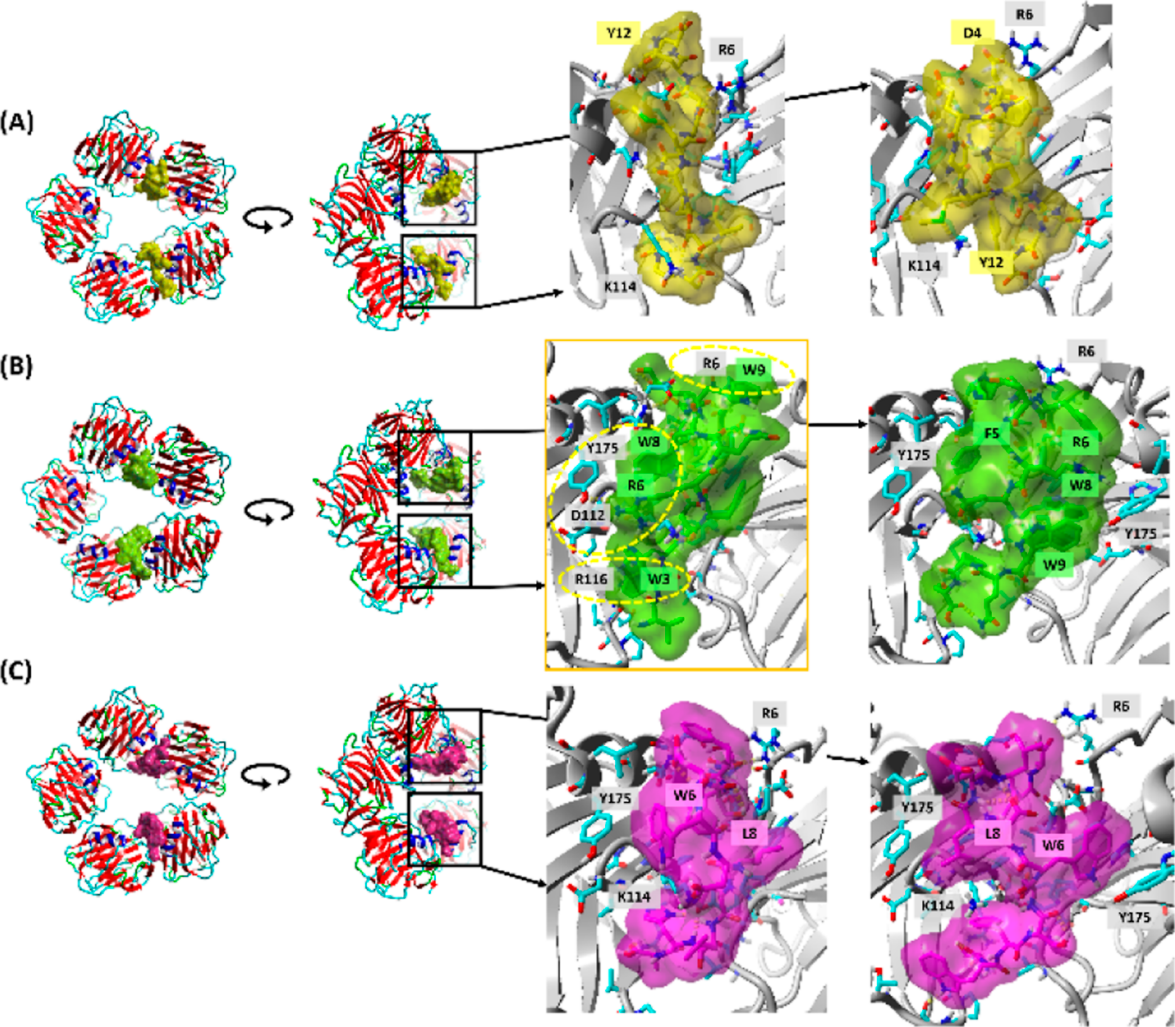
Binding modes of peptides to CRP. (A) P2, (B) P3, and (C) P9. The best antiparallel poses of peptides were selected, and they are magnified in the right panels. The transparent surfaces and the residue labels of selected peptides are colored yellow, green, and violet, respectively. The secondary structure of CRP is shown in gray on the right panels. The orange frame marks the best pose. The most characteristic interactions for this pose are shown as yellow dashed areas.

All investigated peptides preferred to bind to the CRP pentamer in the ridges between the monomers, however, with different conformations, even for the same peptides. The calcium ions removed from the crystal structure before docking are bound far from interfaces between monomers; therefore, they would not influence the binding of peptides. To visualize the best binding poses, we show two of them. These poses are bound in antiparallel mode (Figure 2). Regardless of the different antiparallel ways of binding, the calculated binding energies are roughly the same for the same peptides. Results for two of four tested pockets with the best (the lowest) binding energies for all three peptides are shown in Table S1.

Peptide P2 has the poorest affinity for CRP binding, with binding energies of −143.1 and −138.7 kcal mol^–1^ for the antiparallel poses. Peptide P9 creates complexes with better binding energy: −162.6 and −160.5 kcal mol^–1^, while the binding of peptide P3 is the strongest: −174.4 and −173.9 kcal mol^–1^ (Table S1). Pose no. 1 of peptide P3 is characterized by the lowest energy of binding, the highest surface in contact with the CRP protein (*S*^int.total^), and the highest hydrophobic surface in contact with CRP (*S*^int.hfob^). It means that the highest contact area leads to the best binding, which is justified by the high ratio (*S*^int.hfob^/*S*^int.total^) for all investigated peptides since the interfaces between CRP and peptides are highly hydrophobic.

The peptides bind to the CRP pentamer close to the interior of the pentamer ring and perpendicularly to the ring circle. They bind to the concave surfaces, which are located between CRP monomers, where there are the largest number of residue-reside contacts. Since the interface between CRP monomers is not symmetrical, the antiparallel binding of peptides creates different contacts with CRP. For the best binding, the highest contact surface and many hydrophobic interactions are critical. For all poses, the hydrophobic contribution to the total contact surface is very high and about 90% (Table S1). For the best pose of the best binding peptide P3 (marked by the orange frame in Figure 2B), the most characteristic interactions are shown in yellow dashed areas: (i) π–π face-to-face stacking interaction of CRP:R6 and peptide P3:W9; (ii) residues CRP:D112,Y175 and peptide P3:R6,W8 creating many ionic, hydrogen bonds and π–π stacking interactions; and (iii) π–π face-to-face stacking interaction of CRP:R116 and peptide P3:W3. A large number of aromatic residues of peptide P3, especially tryptophan residues, significantly contribute to its most robust binding to CRP.

### PM-IRRAS Analysis

**Figure 3:**
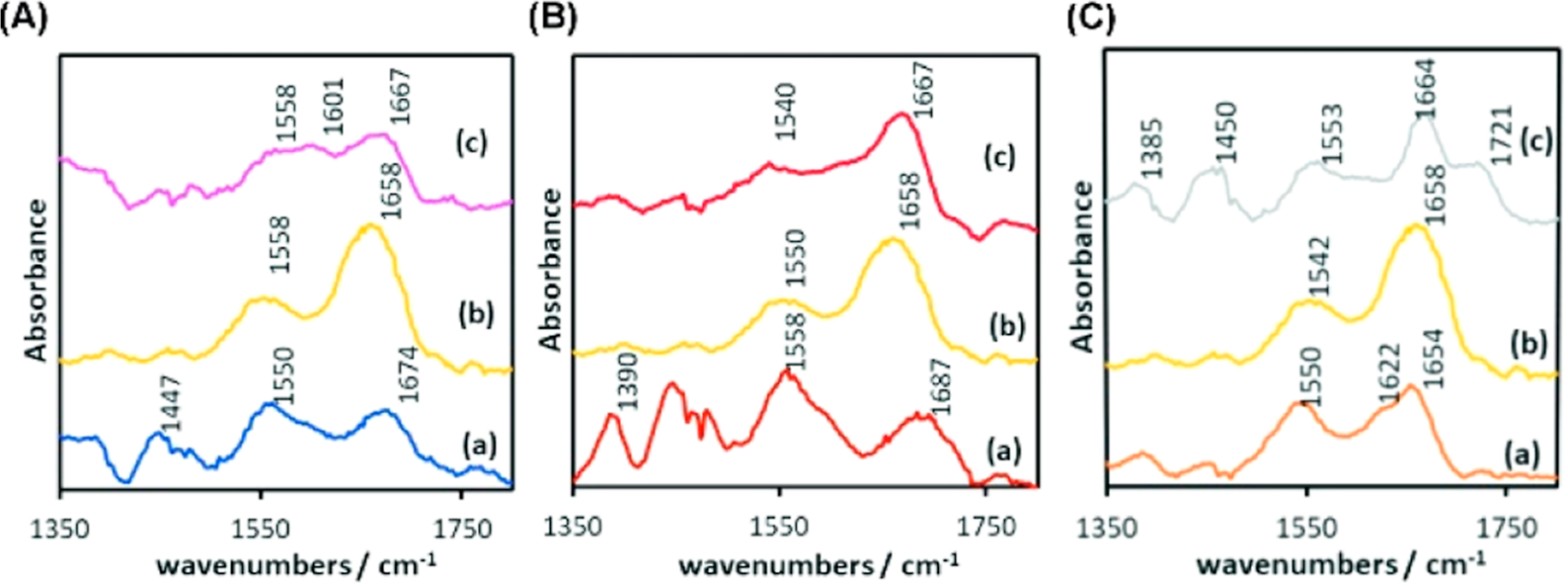
PM-IRRAS spectra of the peptide: (A) P2, (B) P3, and (C) P9; (a) peptides, (b) CRP, and (c) the protein–peptide (P2, P3, and P9) complex immobilized on the gold plates.

The interaction of the immobilized peptides P2, P3, and P9 with CRP was characterized by PM-IRRAS (Figure 3). Figure 3C compares the spectra of the P9 layer on the gold surface to the CRP layer and the P9-CRP complex. The P9 spectrum shows bands characteristic for peptides at 1654, 1622, 1550, 1450, and 1385 cm^–1^.^55^ The bands at 1654 and 1622 cm^–1^ correspond to the amide I mode of the peptide bond connecting the neighboring amino acids. The amide I mode involves contribution from the C=O stretching mode mainly. The position of the amide I band is directly related to the secondary structure of peptides and proteins.^56^ In the spectrum of P9 (Figure 3C(a)), the amide I band is very broad, showing a maximum at 1654 cm^–1^ and a weaker shoulder at 1622 cm^–1^. Typically, the band at 1654 cm^–1^ indicates a rather helical structure of the peptide, but it may also be attributed to the unordered peptide chains.^57^ The band at 1622 cm^–1^ signifies the presence of β-sheets. This result confirms the TEM, where the images obtained for the P9 peptide (Figure 1C) show the structure of the so-called twisted nanoribbons, typical for peptides with a beta-sheet structure.^54^

The band at 1550 cm^–1^ corresponds to the amide II mode involving the N–H bending oscillation. In the spectrum of P9 (Figure 3C(a)), the amide II band is very broad, corroborating the suggestion that P9 adsorbed on gold contains β and α or disordered peptide chains. The amide I band and amide II band are very broad in the CRP spectrum Figure 3C(b), reflecting the complex structure of this protein. The amide I for human CRP at 1632 cm^–1^ with a shoulder at 1652 cm^–1^,^58^ which agrees with the high content of the β structure. The spectrum of CRP adsorbed on gold (Figure 3C(b)) indicates that the structure is strongly affected by the interaction of the protein with the gold support. The content of the helical structures is increased.

The spectrum of the P9-CRP complex differs significantly from the P9 and CRP spectra. For example, in the case of the P9-CRP complex, the new band at 1721 cm^–1^ is observed. Also, the amide I and II bands are shifted compared with CRP and P9. There is also increased absorption in the gap between the amide I and amide II bands. Such extensive changes suggest that the secondary structure of the complex may be affected. The new band at 1721 cm^–1^ suggests the presence of protonated carboxylic groups in the P9-CRP complex structure. Similar spectral features like the new band above 1700 cm^–1^ and the blue shift of the amide I band were observed, e.g., for laccase adsorbed on the gold surface.^59^ The changes were attributed to relaxation of the tertiary structure.

The bands at 1450 and 1385 cm^–1^ originate from the protein side groups. The intensity of these bands is visibly higher in the spectrum of the complex compared to P9 and CRP. Due to the surface selection rule, only oscillations perpendicular to the gold support contribute to the PM-IRRAS spectrum.^60^ The change in relative intensities of the 1450 and 1385 cm^–1^ bands in PM-IRRAS spectra suggests the different orientations of the side groups with respect to the gold surface.

Figure 3A,B presents the results for P2 and P3 peptides and their complexes with CRP, respectively. The peptide P2 immobilized on the gold surface shows very broad amide I and amide II bands—similar to P9. The differences between the two peptides include the position of the amide I band: 1654 and 1622 cm^–1^ for P9 and 1674 cm^–1^ in the case of P2. The relatively high position of the amide I maximum suggests a higher amount of disordered structures in the secondary structure of the immobilized P2. Another difference between P9 and P2 spectra is the low intensity of the amide I band compared with the amide II band (1550 cm^–1^). The amide I and amide II modes involve C=O stretching and N–H bending motions, which are perpendicular to each other. Therefore, the amide I to amide II relative intensity depends on the orientation of the peptide molecule with respect to the gold.^48,61^ If the peptide structure is a single α-helix or single β-sheet, the orientation of the peptide chain with respect to the gold surface can be calculated.^62,63^ Assuming that P2 has helical conformation on the surface, the axis of the helix is nearly parallel to the gold surface, while P9 is tilted with the degree between the axis of the helix and the surface about 48°. Significant changes are visible if the P2, CRP, and P2-CRP complex spectra are compared (Figure 3A). The new spectral component is visible at 1601 cm^–1^. Furthermore, the position of the amide I band of the P2-CRP falls between that of P2 (1674 cm^–1^) and CRP (1658 cm^–1^). There is no component at 1721 cm^–1^, as observed in the case of the P9-CRP complex. The observed changes suggest that the P2-CRP complex is formed, but the effect of the P2 on the CRP secondary structure is different compared to P9. Such an observation agrees with the theoretical prediction (Figure 2). It might also be rationalized by the different orientations of the peptide chain with respect to the gold surface.

The spectrum of P3 immobilized on gold shows the low intensity of the amide I band—similar to the P2 spectrum. Assuming the helical conformation of P3 at the gold surface, like for the other two peptides, the angle between the helical axis and normal to the surface equals 35°. The P3 molecules are thus tilted similarly to P9. The position of the amide I band is significantly blue-shifted—to 1687 cm^–1^ compared with P2 and P9, suggesting the presence of disordered structures. The spectrum of the P3-CRP complex resembles the spectrum of CRP, though the position of the amide I band is shifted. The similarity between the CRP and P3-CRP complex suggests that the secondary structure of CRP is preserved in the complex. The spectrum of the P3-CRP complex is relatively intense compared to P9-CRP and P2-CRP, suggesting that a relatively high amount of the protein binds to P3. This result is also supported by previous studies (Figures S1, and 2).

In summary, PM-IRRAS results suggest that P2, P3, and P9 peptides attain various orientations with respect to the gold surface. The interaction with CRP is visible in all three cases, but the secondary structures of the peptide–CRP complex are different. The differences result probably from that the three peptides interact with slightly other groups/amino acids of CRP—as visualized in Figure 2. The various orientations of peptides contribute to the peptide–CRP interaction as well.

### Electrochemical Recognition of CRP at Peptide-Modified Interfaces

**Figure 4:**
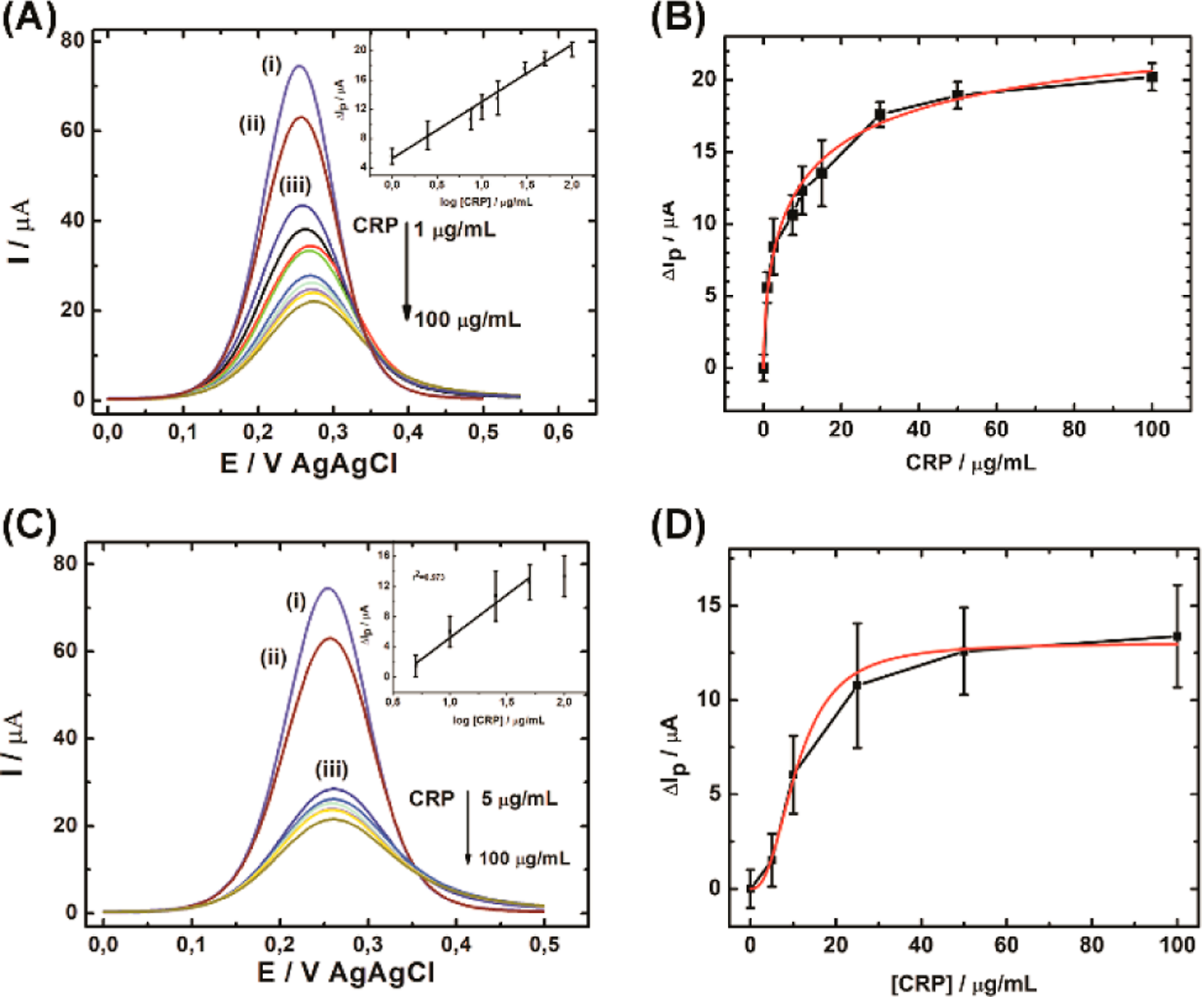
DPV voltammograms obtained for (i) bare ITO (ii) ITO/TESPSA (iii) ITO/TESPSA/P3 or mAb; (A) P3 peptide or (C) anti-CRP antibodies, and subsequent DPV curves obtained for different CRP concentrations for ITO/TESPSA/P3 or mAb in 1 mM Fc(CH_2_OH)_2_/PBS. Inset; Δ*I*_p_ vs log. concentration of CRP obtained for ITO/TESPSA/P3 (A) and ITO/TESPSA/mAb (C). Hill plots showing Δ*I*_p_ vs. CRP concentration used for the determination of *K*_d_ toward CRP for (B) P3 peptide and (D) mAb. For fitted values see the main text.

As shown above, the P3 peptide has the highest affinity for CRP among the studied peptides. Therefore, it was chosen to recognize CRP at a silicate-modified ITO. The salinization of the ITO with TESPSA was used to generate functional groups (–COOH), allowing the covalent binding of the P3 peptide (see Section 3.1 in the Supporting Information). Molecular recognition of the CRP with concentrations ranging from 1.0 to 100 μg mL^–1^ by the P3-peptide was examined on these modified electrodes. Figure 4A shows DPV curves recorded for all stages of electrode modification with the P3-peptide and in the presence of 1.0–100 μg mL^–1^ of CRP (Figure 4A). For comparison, the ITO/TESPSA was modified with commercially available anti-CRP mAb, and the obtained electrode was measured in the presence of CRP (5.0–100 μg mL^–1^) (Figure 4C). Based on the recorded curves, a decrease in the peak current can be observed along with the subsequent stages of electrode modification with TESPSA, P3 peptide/mAb assembly, and CRP recognition (Figure 4A,C). The peak current for ITO/TESPSA/P3 decreases as the CRP concentration increases. The drop of the peak current with subsequent modification of the ITO electrode, and the addition of the CRP, result from the hindered access of the redox molecules to the electrode surface by the hydrophobic and blocking layers of silicate/protein. These studies demonstrated that CRP is recognized by the P3 peptide immobilized on TESPSA-modified ITO. The decrease in the peak current (Δ*I*_p_, defined as the difference from the SWV with [CRP] = 0) varies linearly with the logarithm of the CRP concentration (Figure 4A inset). The obtained concentration range (1.0–100 μg mL^–1^) with a detection limit (LOD) (LOD = 3σ/*S*, where σ is the standard deviation of the blank (ITO/TESPSA/P3 or mAb without CRP), and *S* is the slope of the linear region of the calibration curve (Figure 4A,C inset) of 0.34 μg mL^–1^ for ITO/TESPSA/P3 is lower than CRP-binding phage-based electrodes^36^ and similar to those obtained for electrodes modified with anti-CRP antibodies,^27,46,64^ and aptamers^33^ that have been presented by others (Table S2). However, the antibody electrode presented in this article has a significantly higher LOD (3.6 μg mL^–1^) and linear response range from 5 to 50 μg mL^–1^ (Figure 4C inset), which shows that the ITO/TESPSA/mAb electrode is far from the optimized design. Also, it can be noticed that at a concentration of 100 μg mL^–1^, the response recorded for the antibody electrode (ITO/TESPSA/mAb Figure 4C) is not so clear, which results from the lack of accessibility from the binding sides (saturation state). However, the peptide-based electrode successfully recognizes the CRP in a range that would enable the differentiation of viral from bacterial infections (0–50 μg mL^–1^). The dissociation constants for both the antibody and the peptide were evaluated using the Hill equation (eq 1)^65^
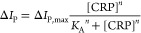
1where *K*_A_ is the CRP concentration at half the maximum current difference, and *n* is the Hill coefficient (Figure 4B,D). The equilibrium dissociation constant is given by *K*_d_ = *K*_A_^*n*^. In the case of the anti-CRP mAb, *K*_d_ = 336 ± 144 μg mL^–1^ (2.8 ± 1.2 μM), with a Hill coefficient of 2.4 ± 0.3 (Figure 4D).

The obtained value of *K*_d_ is comparable with the other platforms modified with anti-CRP antibodies;^30,44^ e.g., the dissociation constant for the P3 peptide is significantly lower, at 4.2 ± 0.144 μg mL^–1^ (35 ± 1.2 nM) (Figure 4B). The Hill coefficient is 0.6, indicating a negative cooperativity. One reason can be the higher surface density of the peptide as compared to the antibodies, which can lead to increased steric hindrance.^66^ The obtained value of *K*_d_ for P3 peptide is comparable with the other platforms modified with aptamers^35^ or nanobodies,^40^ and lower for anti-CRP antibodies-based systems.^30,44^ In the case of mAb, the *K*_d_ value is relatively uncertain because of the large error bars. But the shape of the curve clearly indicates a *n* larger than 1.

The selectivity of ITO/TESPSA/P3 was evaluated in the presence of three interfering proteins: TnT (a marker of cardiac disease) and Fib (one of the acute-phase proteins), (Il-6) (one of the inflammatory cytokine). The results are depicted in Figure S3. The clear drop of the current was visible after ITO/TESPSA/P3 incubation with 2.5 μg mL^–1^ of CRP. When the electrode was incubated with the 2.5 μg mL^–1^ of TnT, IL-6, and Fib separately, a higher peak current of ca. 20–25% was recorded compared to CRP. Moreover, the recorded peak current for ITO/TESPSA/P3 electrodes in the presence of TnT and Fib is comparable with the peak current obtained for ITO/TESPSA/P3 electrodes in the absence of proteins, indicating a lack of specific interactions. The outcomes of this study show that ITO/TESPSA/P3 recognizes the CRP while it is not specific for the TnT and Fib, thereby showing that the P3 peptide exhibits a good selectivity toward CRP.

## Conclusions

In this work, we identified and synthesized the three most promising CRP-binding peptides derived from a phage library. The results based on plaque and ELISA tests show that the P3 clone exhibits the highest affinity toward CRP, which is 2 orders of magnitude higher than the wild-type/non-CRP binder. The diversity of the amino acid sequences on the exposed peptides impacts their structure formation from typical linear to helical-like structures. This change was also supported by the PM-IRRAS analysis. The obtained data show that the spectrum of the P3-CRP complex is relatively intense compared to those of P9-CRP and P2-CRP, suggesting that a high amount of the protein binds to P3.

The P3 peptide was successfully applied for silicate-based modification of the ITO electrode to recognize CRP electrochemically. The obtained parameters: operation range of concentration for the P3 peptide-based electrode is 1.0–100 μg mL^–1^ (with a detection limit of 0.34 μg mL^–1^), and *K*_d_ of 4.2 ± 0.144 μg mL^–1^, were compared with the antibody-based electrode used as a reference system. The results show that P3 peptide-based electrode exhibits lower detection limits and *K*_d_ than antibody-based electrodes. The presented immobilization protocol is simple and electrochemical measurements could be a way to reach a quick conclusion about binding affinity. In comparison, the *K*_d_ evaluated from the fluorescent or surface plasmon measurements would not be very informative, in terms of the development of the electrochemical sensor.

The P3 peptide-based electrode is selective against TnT, Fib, and IL-6. These results demonstrate that the presented P3 peptide is an alternative to antibodies as a basis for constructing new, stable, cheap, and selective sensor platforms for CRP. The operation range of the new electrode is such that it can be used to differentiate viral from bacterial infection.

While there are several biosensors in the literature whose LODs are lower and their linear range responses wider than those obtained for our peptide-based electrode, we describe a new approach to sensor development. The added value of this work is the integration of experimental methods with computational modeling analysis. The modeling from known amino acid sequences of peptides confirms that the P3 peptide is the best binder for CRP. Such a combined approach has not been reported previously and demonstrates how numerical methods/in silico analysis can replace or enhance laborious experimental techniques. Using molecular docking to identify the best binders eliminates the application of chemicals, which is vital to developing greener chemistry. This study validates the numerical approach to identifying peptide binding properties and represents an important step on the road to peptide-based sensors. Moreover, if we know how the docking works, in the future, we could tailor the sequence of the peptide by changing, for example, one of the amino acids. Then, using artificial intelligence algorithms^9^ to calculate the binding energy for the new sequence, and if the score is high, we could synthesize a new peptide and use it to recognize the target.

## Data Availability

Data are available in the repository.^67^

## Supporting Information Available

Experimental section and additional experiments (PDF)

## References

[ref1] Lv M.; Jan Cornel E.; Fan Z.; Du J. Advances and Perspectives of Peptide and Polypeptide-Based Materials for Biomedical Imaging. Adv. NanoBiomed Res. 2021, 1, 2000109 10.1002/anbr.202000109.

[ref2] Qi G.-B.; Gao Y.-J.; Wang L.; Wang H. Self-Assembled Peptide-Based Nanomaterials for Biomedical Imaging and Therapy. Adv. Mater. 2018, 30, 1703444 10.1002/adma.201703444. 29460400

[ref3] Akhavan O.; Ghaderi E. Graphene Nanomesh Promises Extremely Efficient In Vivo Photothermal Therapy. Small 2013, 9, 3593–3601. 10.1002/smll.201203106. 23625739

[ref4] Petrenko V. A.; Vodyanoy V. J. Phage Display for Detection of Biological Threat Agents. J. Microbiol. Methods 2003, 53, 253–262. 10.1016/S0167-7012(03)00029-0. 12654496

[ref5] Vanova V.; Mitrevska K.; Milosavljevic V.; Hynek D.; Richtera L.; Adam V. Peptide-Based Electrochemical Biosensors Utilized for Protein Detection. Biosens. Bioelectron. 2021, 180, 113087 10.1016/j.bios.2021.113087. 33662844

[ref6] Huan Y.; Kong Q.; Mou H.; Yi H. Antimicrobial Peptides: Classification, Design, Application and Research Progress in Multiple Fields. Front. Microbiol. 2020, 11, 2559 10.3389/fmicb.2020.582779. PMC7596191 33178164

[ref7] Tanaka T.; Kokuryu Y.; Matsunaga T. Novel Method for Selection of Antimicrobial Peptides from a Phage Display Library by Use of Bacterial Magnetic Particles. Appl. Environ. Microbiol. 2008, 74, 7600–7606. 10.1128/AEM.00162-08. 18952877 PMC2607169

[ref8] Wu K.; Bai H.; Chang Y.-T.; Redler R.; McNally K. E.; Sheffler W.; Brunette T. J.; Hicks D. R.; Morgan T. E.; Stevens T. J.; Broerman A.; Goreshnik I.; DeWitt M.; Chow C. M.; Shen Y.; Stewart L.; Derivery E.; Silva D. A.; Bhabha G.; Ekiert D. C.; Baker D. De Novo Design of Modular Peptide-Binding Proteins by Superhelical Matching. Nature 2023, 616, 581–589. 10.1038/s41586-023-05909-9. 37020023 PMC10115654

[ref9] Szymczak P.; Możejko M.; Grzegorzek T.; Jurczak R.; Bauer M.; Neubauer D.; Sikora K.; Michalski M.; Sroka J.; Setny P.; Kamysz W.; Szczurek E. Discovering Highly Potent Antimicrobial Peptides with Deep Generative Model HydrAMP. Nat. Commun. 2023, 14, 1453 10.1038/s41467-023-36994-z. 36922490 PMC10017685

[ref10] Chang L.; Perez A. Ranking Peptide Binders by Affinity with AlphaFold**. Angew. Chem., Int. Ed. 2023, 62, e202213362 10.1002/anie.202213362. 36542066

[ref11] Ramakrishnan M.; van Teijlingen A.; Tuttle T.; Ulijn R. V. Integrating Computation, Experiment, and Machine Learning in the Design of Peptide-Based Supramolecular Materials and Systems. Angew. Chem., Int. Ed. 2023, 62, e202218067 10.1002/anie.202218067. 36725681

[ref12] Banta S.; Dooley K.; Shur O. Replacing Antibodies: Engineering New Binding Proteins. Annu. Rev. Biomed. Eng. 2013, 15, 93–113. 10.1146/annurev-bioeng-071812-152412. 23642248

[ref13] Park J. P.; Cropek D. M.; Banta S. High Affinity Peptides for the Recognition of the Heart Disease Biomarker Troponin I Identified Using Phage Display. Biotechnol. Bioeng. 2009, 105, 678–686. 10.1002/bit.22597. 19891006

[ref14] Padmanaban G.; Park H.; Choi J. S.; Cho Y. W.; Kang W. C.; Moon C. I.; Kim I. S.; Lee B. H. Identification of Peptides That Selectively Bind to Myoglobin by Biopanning of Phage Displayed-Peptide Library. J. Biotechnol. 2014, 187, 43–50. 10.1016/j.jbiotec.2014.07.435. 25078431

[ref15] Chung W. J.; Oh J. W.; Kwak K.; Lee B. Y.; Meyer J.; Wang E.; Hexemer A.; Lee S. W. Biomimetic Self-Templating Supramolecular Structures. Nature 2011, 478, 364–368. 10.1038/nature10513. 22012394

[ref16] Pavan S.; Berti F. Short Peptides as Biosensor Transducers. Anal. Bioanal. Chem. 2012, 402, 3055–3070. 10.1007/s00216-011-5589-8. 22169951

[ref17] Kim J. H.; Cho C. H.; Shin J. H.; Hyun M. S.; Hwang E.; Park T. J.; Park J. P. Biomimetic Isolation of Affinity Peptides for Electrochemical Detection of Influenza Virus Antigen. Sens. Actuators, B 2021, 343, 130161 10.1016/j.snb.2021.130161.

[ref18] Zhang J.; Spring H.; Schwab M. Neuroblastoma Tumor Cell-Binding Peptides Identified through Random Peptide Phage Display. Cancer Lett. 2001, 171, 153–164. 10.1016/S0304-3835(01)00575-4. 11520599

[ref19] Wu J.; Cropek D. M.; West A. C.; Banta S. Development of a Troponin i Biosensor Using a Peptide Obtained through Phage Display. Anal. Chem. 2010, 82 (19), 8235–8243. 10.1021/ac101657h. 20831206

[ref20] Lee H. Y.; Choi J. S.; Guruprasath P.; Lee B.-H.; Cho Y. W. An Electrochemical Biosensor Based on a Myoglobin-Specific Binding Peptide for Early Diagnosis of Acute Myocardial Infarction. Anal. Sci. 2015, 31, 699–704. 10.2116/analsci.31.699. 26165294

[ref21] Tillett W. S.; Francis T. Serological Reactions In Pneumonia With A Nonprotein Somatic Fraction Of Pneumococcus. J. Exp. Med. 1930, 52, 561–571. 10.1084/jem.52.4.561. 19869788 PMC2131884

[ref22] Ngwa D. N.; Agrawal A. Structure-Function Relationships of C-Reactive Protein in Bacterial Infection. Front. Immunol. 2019, 10, 166 10.3389/fimmu.2019.00166. 30863393 PMC6400226

[ref23] Sproston N. R.; Ashworth J. J. Role of C-Reactive Protein at Sites of Inflammation and Infection. Front. Immunol. 2018, 9, 754 10.3389/fimmu.2018.00754. 29706967 PMC5908901

[ref24] Bryan T.; Luo X.; Bueno P. R.; Davis J. J. An Optimised Electrochemical Biosensor for the Label-Free Detection of C-Reactive Protein in Blood. Biosens. Bioelectron. 2013, 39, 94–98. 10.1016/j.bios.2012.06.051. 22809521

[ref25] Hart P. C.; Rajab I. M.; Alebraheem M.; Potempa L. A. C-Reactive Protein and Cancer—Diagnostic and Therapeutic Insights. Front. Immunol. 2020, 11, 1–17. 10.3389/fimmu.2020.595835. 33324413 PMC7727277

[ref26] Kim K.-W.; Kim B.-M.; Moon H.-W.; Lee S.-H.; Kim H.-R. Role of C-Reactive Protein in Osteoclastogenesis in Rheumatoid Arthritis. Arthritis Res. Ther. 2015, 17, 41 10.1186/s13075-015-0563-z. 25889630 PMC4372175

[ref27] Sonuç Karaboğa M. N.; Sezgintürk M. K. A Novel Silanization Agent Based Single Used Biosensing System: Detection of C-Reactive Protein as a Potential Alzheimer’s Disease Blood Biomarker. J. Pharm. Biomed. Anal. 2018, 154, 227–235. 10.1016/j.jpba.2018.03.016. 29558723

[ref28] Ali N. Elevated Level of C-Reactive Protein May Be an Early Marker to Predict Risk for Severity of COVID-19. J. Med. Virol. 2020, 92, 2409–2411. 10.1002/jmv.26097. 32516845 PMC7301027

[ref29] Wang W.; Mai Z.; Chen Y.; Wang J.; Li L.; Su Q.; Li X.; Hong X. A Label-Free Fiber Optic SPR Biosensor for Specific Detection of C-Reactive Protein. Sci. Rep. 2017, 7, 16904 10.1038/s41598-017-17276-3. 29203814 PMC5715095

[ref30] Meyer M. H. F.; Hartmann M.; Keusgen M. SPR-Based Immunosensor for the CRP Detection—A New Method to Detect a Well Known Protein. Biosens. Bioelectron. 2006, 21, 1987–1990. 10.1016/j.bios.2005.09.010. 16246542

[ref31] Raveendran M.; Lee A. J.; Sharma R.; Wälti C.; Actis P. Rational Design of DNA Nanostructures for Single Molecule Biosensing. Nat. Commun. 2020, 11, 4384 10.1038/s41467-020-18132-1. 32873796 PMC7463249

[ref32] Piccoli J.; Hein R.; El-Sagheer A. H.; Brown T.; Cilli E. M.; Bueno P. R.; Davis J. J. Redox Capacitive Assaying of C-Reactive Protein at a Peptide Supported Aptamer Interface. Anal. Chem. 2018, 90, 3005–3008. 10.1021/acs.analchem.7b05374. 29411973

[ref33] Centi S.; Bonel Sanmartin L.; Tombelli S.; Palchetti I.; Mascini M. Detection of C Reactive Protein (CRP) in Serum by an Electrochemical Aptamer-Based Sandwich Assay. Electroanalysis 2009, 21, 1309–1315. 10.1002/elan.200804560.

[ref34] Li H.-H.; Wen C.-Y.; Hong C.-Y.; Lai J.-C. Evaluation of Aptamer Specificity with or without Primers Using Clinical Samples for C-Reactive Protein by Magnetic-Assisted Rapid Aptamer Selection. RSC Adv. 2017, 7, 42856–42865. 10.1039/C7RA07249J.

[ref35] Pohanka M. Diagnoses Based on C-Reactive Protein Point-of-Care Tests. Biosensors 2022, 12, 344 10.3390/bios12050344. 35624645 PMC9138282

[ref36] Szot-Karpińska K.; Kudła P.; Szarota A.; Narajczyk M.; Marken F.; Niedziółka-Jönsson J. CRP-Binding Bacteriophage as a New Element of Layer-by-Layer Assembly Carbon Nanofiber Modified Electrodes. Bioelectrochemistry 2020, 136, 107629 10.1016/j.bioelechem.2020.107629. 32818758

[ref37] Al-Enezi E.; Vakurov A.; Eades A.; Ding M.; Jose G.; Saha S.; Millner P. Affimer-Based Europium Chelates Allow Sensitive Optical Biosensing in a Range of Human Disease Biomarkers. Sensors 2021, 21, 831 10.3390/s21030831. 33513673 PMC7865513

[ref38] Yang H. J.; Kim M. W.; Raju C. V.; Cho C. H.; Park T. J.; Park J. P. Highly Sensitive and Label-Free Electrochemical Detection of C-Reactive Protein on a Peptide Receptor–gold Nanoparticle–black Phosphorous Nanocomposite Modified Electrode. Biosens. Bioelectron. 2023, 234, 115382 10.1016/j.bios.2023.115382. 37178497

[ref39] Bessette P. H.; Rice J. J.; Daugherty P. S. Rapid Isolation of High-Affinity Protein Binding Peptides Using Bacterial Display. Protein Eng. Des. Sel. 2004, 17, 731–739. 10.1093/protein/gzh084. 15531628

[ref40] Oloketuyi S.; Bernedo R.; Christmann A.; Borkowska J.; Cazzaniga G.; Schuchmann H. W.; Niedziółka-Jönsson J.; Szot-Karpińska K.; Kolmar H.; de Marco A. Native Llama Nanobody Library Panning Performed by Phage and Yeast Display Provides Binders Suitable for C-Reactive Protein Detection. Biosensors 2021, 11, 496 10.3390/bios11120496. 34940253 PMC8699515

[ref41] Zhang L.; Li H. Y.; Li W.; Shen Z. Y.; Wang Y. D.; Ji S. R.; Wu Y. An ELISA Assay for Quantifying Monomeric C-Reactive Protein in Plasma. Front. Immunol. 2018, 9, 511 10.3389/fimmu.2018.00511. 29593741 PMC5857914

[ref42] Vashist S. K.; Venkatesh A. G.; Marion Schneider E.; Beaudoin C.; Luppa P. B.; Luong J. H. T. Bioanalytical Advances in Assays for C-Reactive Protein. Biotechnol. Adv. 2016, 34, 272–290. 10.1016/j.biotechadv.2015.12.010. 26717866

[ref43] Hwang A.; Kim E.; Moon J.; Lee H.; Lee M.; Jeong J.; Lim E.-K.; Jung J.; Kang T.; Kim B. Atomically Flat Au Nanoplate Platforms Enable Ultraspecific Attomolar Detection of Protein Biomarkers. ACS Appl. Mater. Interfaces 2019, 11, 18960–18967. 10.1021/acsami.9b04363. 31062578

[ref44] Aray A.; Chiavaioli F.; Arjmand M.; Trono C.; Tombelli S.; Giannetti A.; Cennamo N.; Soltanolkotabi M.; Zeni L.; Baldini F. SPR-Based Plastic Optical Fibre Biosensor for the Detection of C-Reactive Protein in Serum. J. Biophot. 2016, 9, 1077–1084. 10.1002/jbio.201500315. 27089540

[ref45] Thangamuthu M.; Santschi C.; J F Martin O. Label-Free Electrochemical Immunoassay for C-Reactive Protein. Biosensors 2018, 8, 34 10.3390/bios8020034. 29601504 PMC6022967

[ref46] Kim G. W.; Zheng S.; Kim M. S.; Cheon S. A.; Ko S.; Park T. J. Development of Specific Immobilization Method on Gold Surface and Its Application for Determining Cardiac Risk. BioChip J. 2014, 8, 295–302. 10.1007/s13206-014-8408-4.

[ref47] Kowalczyk A.; Sęk J. P.; Kasprzak A.; Poplawska M.; Grudzinski I. P.; Nowicka A. M. Occlusion Phenomenon of Redox Probe by Protein as a Way of Voltammetric Detection of Non-Electroactive C-Reactive Protein. Biosens. Bioelectron. 2018, 117, 232–239. 10.1016/j.bios.2018.06.019. 29908448

[ref48] Piccoli J. P.; Soares A. C.; Oliveira O. N.; Cilli E. M. Nanostructured Functional Peptide Films and Their Application in C-Reactive Protein Immunosensors. Bioelectrochemistry 2021, 138, 107692 10.1016/j.bioelechem.2020.107692. 33291002

[ref49] Piestrzyńska M.; Dominik M.; Kosiel K.; Janczuk-Richter M.; Szot-Karpińska K.; Brzozowska E.; Shao L.; Niedziółka-Jonsson J.; Bock W. J.; Śmietana M. Ultrasensitive Tantalum Oxide Nano-Coated Long-Period Gratings for Detection of Various Biological Targets. Biosens. Bioelectron. 2019, 133, 8–15. 10.1016/j.bios.2019.03.006. 30903939

[ref50] Guillon C.; Bigouagou U.; Folio C.; Jeannin P.; Delneste Y.; Gouet P. A Staggered Decameric Assembly of Human C-Reactive Protein Stabilized by Zinc Ions Revealed by X-Ray Crystallography. Protein Pept. Lett. 2015, 22, 248–255. 10.2174/0929866522666141231111226. 25552313

[ref51] An J.; Totrov M.; Abagyan R. Pocketome via Comprehensive Identification and Classification of Ligand Binding Envelopes *. Mol. Cell. Proteomics 2005, 4, 752–761. 10.1074/mcp.M400159-MCP200. 15757999

[ref52] Abagyan R.; Totrov M. Biased Probability Monte Carlo Conformational Searches and Electrostatic Calculations for Peptides and Proteins. J. Mol. Biol. 1994, 235, 983–1002. 10.1006/jmbi.1994.1052. 8289329

[ref53] Sawada T. Filamentous Virus-Based Soft Materials Based on Controlled Assembly through Liquid Crystalline Formation. Polym. J. 2017, 49, 639–647. 10.1038/pj.2017.35.

[ref54] Song S.; Wang J.; Song N.; Di H.; Liu D.; Yu Z. Peptide Interdigitation-Induced Twisted Nanoribbons as Chiral Scaffolds for Supramolecular Nanozymes. Nanoscale 2020, 12, 2422–2433. 10.1039/C9NR09492J. 31916547

[ref55] Tammer M. G. Sokrates: Infrared and Raman Characteristic Group Frequencies: Tables and Charts. Colloid Polym. Sci. 2004, 283 (2), 235 10.1007/s00396-004-1164-6.

[ref56] Miyazawa T.; Blout E. R. The Infrared Spectra of Polypeptides in Various Conformations: Amide I and II Bands1. J. Am. Chem. Soc. 1961, 83, 712–719. 10.1021/ja01464a042.

[ref57] Ragusa S.; Cambria M. T.; Pierfederici F.; Scirè A.; Bertoli E.; Tanfani F.; Cambria A. Structure–Activity Relationship on Fungal Laccase from Rigidoporus Lignosus: A Fourier-Transform Infrared Spectroscopic Study. Biochim. Biophys. Acta, Proteins Proteomics 2002, 1601, 155–162. 10.1016/S1570-9639(02)00469-7. 12445477

[ref58] Andersson P. O.; Viberg P.; Forsberg P.; Nikolajeff F.; Österlund L.; Karlsson M. Nanocrystalline Diamond Sensor Targeted for Selective CRP Detection: An ATR-FTIR Spectroscopy Study. Anal. Bioanal. Chem. 2016, 408, 3675–3680. 10.1007/s00216-016-9485-0. 27007740

[ref59] Olejnik P.; Pawłowska A.; Pałys B. Application of Polarization Modulated Infrared Reflection Absorption Spectroscopy for Electrocatalytic Activity Studies of Laccase Adsorbed on Modified Gold Electrodes. Electrochim. Acta 2013, 110, 105–111. 10.1016/j.electacta.2013.03.089.

[ref60] Greenler R. G. Infrared Study of Adsorbed Molecules on Metal Surfaces by Reflection Techniques. J. Chem. Phys. 1966, 44, 310–315. 10.1063/1.1726462.

[ref61] Olejnik P.; Palys B.; Kowalczyk A.; Nowicka A. M. Orientation of Laccase on Charged Surfaces. Mediatorless Oxygen Reduction on Amino- and Carboxyl-Ended Ethylphenyl Groups. J. Phys. Chem. C 2012, 116, 25911–25918. 10.1021/jp3098654.

[ref62] Morita T.; Kimura S. Long-Range Electron Transfer over 4 Nm Governed by an Inelastic Hopping Mechanism in Self-Assembled Monolayers of Helical Peptides. J. Am. Chem. Soc. 2003, 125, 8732–8733. 10.1021/ja034872n. 12862461

[ref63] Miura Y.; Kimura S.; Imanishi Y.; Umemura J. Formation of Oriented Helical Peptide Layers on a Gold Surface Due to the Self-Assembling Properties of Peptides. Langmuir 1998, 14, 6935–6940. 10.1021/la981296d.

[ref64] Sonuç Karaboğa M. N.; Sezgintürk M. K. Determination of C-Reactive Protein by PAMAM Decorated ITO Based Disposable Biosensing System: A New Immunosensor Design from an Old Molecule. Talanta 2018, 186, 162–168. 10.1016/j.talanta.2018.04.051. 29784344

[ref65] Gesztelyi R.; Zsuga J.; Kemeny-Beke A.; Varga B.; Juhasz B.; Tosaki A. The Hill Equation and the Origin of Quantitative Pharmacology. Arch. Hist. Exact Sci. 2012, 66, 427–438. 10.1007/s00407-012-0098-5.

[ref66] Jin X.; Talbot J.; Wang N.-H. L. Analysis of Steric Hindrance Effects on Adsorption Kinetics and Equilibria. AIChE J. 1994, 40, 1685–1696. 10.1002/aic.690401010.

[ref67] Szot-Karpińska K. Electrochemical data for electrodes modified with peptides for molecular recognition of C-reactive protein, RepOD, 1 DOI: 10.18150/AWPXYO.

