# Supplementary material for: Investigation of Peptides for Molecular Recognition of C-Reactive Protein–Theoretical and Experimental Studies
Source: Anal Chem. 2023 Sep 11;95(38):14475–83. doi: 10.1021/acs.analchem.3c03127 (PMC10535004; doi:10.1021/acs.analchem.3c03127)
Supplement: Supplementary file 1 — ac3c03127_si_001.pdf [file ac3c03127_si_001.pdf]

Supplementary Information

Investigation of peptides for molecular recognition of C-reactive protein – theoretical and experimental studies

Katarzyna Szot-Karpińska[1*], Patryk Kudła[1†], Urszula Orzeł[2,3], Magdalena Narajczyk[4], Martin Jönsson -Niedziółka[1], Barbara Pałys[3], Sławomir Filipek[2,3], Andreas Ebner[5], Joanna Niedziółka-Jönsson[1]

[1] Institute of Physical Chemistry, Polish Academy of Sciences, Kasprzaka 44/52, 01-224 Warsaw, Poland
[2] Biological and Chemical Research Centre, University of Warsaw, Zwirki i Wigury 101, 02-089 Warsaw, Poland
[3] Faculty of Chemistry, University of Warsaw, Pasteura 1, 02-093 Warsaw, Poland
[4] Department of Electron Microscopy, Faculty of Biology, University of Gdansk, Wita Stwosza 59, 80-308 Gdansk, Poland
[5] Institute of Biophysics, Johannes Kepler University, Gruberstrasse 40, 4020 Linz, Austria

* Corresponding author: kszot@ichf.edu.pl
† Deceased.

Table of contents

1. Materials S2
2. Surface characterization S3
3. Figure S1 and Figure S2 .... S5
4. Figure S3 .... S6
5. Table S1 .... S7
6. Table S2 .... S8

1. Experimental

1.1. Materials

Horseradish peroxidase-conjugated anti-M13pVIII monoclonal antibody was bought from New England Biolabs (NEB, USA) and used in dilution 1:5000, while 3,3',5,5'-tetramethylbenzidine (TMB) was delivered by Thermo Fisher Scientific (USA) and used without pretreatment. Nunc MicroWell 96-Well (Thermo Fisher Scientific). Human C-reactive protein (CRP) and monoclonal antibodies anti-CRP (anti-CRP mAb) were bought from Biorbyt (UK). Fibryno-gen (Fib) from human plasma was purchased from Biosolutions (USA). Bovine serum albumin (BSA), interleukin 6 (IL-6), troponin T (TnT) from human cardiac muscle, PBS tablets (phosphate-buffered saline; 137 mM NaCl, 2.7 mM KCl, and 10 mM phosphate buffer, pH7.4), triethoxysilylpropyl succinic anhydride (TESPSA), Tween 20, $NaHCO_3$ were delivered by Sigma Aldrich. 1,1'-Ferrocenodimethanol ($Fc(CH_2OH)_2$, Acros Organics, Poland. H_2SO_4 96% (Stanlab, Poland). InvitrogenTM DynabeadsTM M _270 Epoxysurface-modified magnetic nanoparticles (mNP) were purchased from Thermo Fischer Scientific (USA). Ph.D.-12 Phage Display Peptide Library, E. coli ER2738, an M13 wild-type bacteriophage (WT) were from NEB (USA).

DNAs were isolated and purified, as described by Wilson [1]. The concentrations of biological samples/DNAs were evaluated using the Thermo Scientific Nano Drop™ One/OneC Microvolume UV-Vis Spectrophotometer. The phage genome sequencing [2] was done by Genomed (Poland) using the 96 gpIII sequencing primer (NEB).

Peptides were synthesised manually by the solid-phase Fmoc/tBu method on 2-chlorotrityl resin (Lipopharm, Poland). The compounds were precipitated with cooled diethyl ether and lyophilised. The compounds were purified by RP-HPLC. Pure fractions (>95%, HPLC) were collected and lyophilised. The identity of all compounds was confirmed by mass spectrometry (ESI-MS).

1.2. CRP binding bacteriophage isolation and characterisation – plaque test

The phage display/affinity selection technique was used to select bacteriophages exposing peptides specific to CRP. The detailed procedure is presented in our previous studies [2]. Briefly, the CRP was bound to the magnetic beads by forming a covalent bond between epoxy and amino groups. Then the phage library was added, so the CRP-binders were binding to the CRP while the other clones were washed away. The bounded phages were then eluted and amplified (phage lysate). The number of binding clones was evaluated using the viral plaque test with blue/white screening. The obtained phage lysate underwent two further biopanning steps to obtain a solution containing only

the phages binding with high affinity to CRP. Ten clones were separated as a single-clone lysate. The binding efficiency was calculated using the plaque assays and was expressed as the ratio O/I, where O denotes the amount of the output phages (eluted phages), and I presents the number of input phages, which were incubated with CRP.

1.3. Screening test of selected CRP-binding phages – enzyme-linked immunosorbent assay (ELISA)

In order to identify the best CRP-binder, the selected clones, in addition to the plaque test, were screened by the ELISA test. This assay was performed as follows: to the wells of the 96-wells plate, 100 µL of CRP (1 µg mL^{-1}) was added. The last three wells were filled with 100 µL of BSA (1 µg mL^{-1}) as a negative control and left overnight at 4 °C for incubation. The wells were then emptied, and 100 µL of 5 mg mL^{-1} BSA in 0.1M $NaHCO_3$ at pH 9.6 was added to the well and incubated for 2h at 4 °C. Subsequently, the wells were emptied, washed four times with PBST (PBS with 0.1% Tween) (0.1 %) via 5 min, filled with 100 µL of phages lysates in 0.1% PBST (P1-P10, and WT as control with concentrations of 10^{11} pfu mL^{-1} – plaque forming unit per mL) and incubated at room temperature (RT). After an hour of incubation, the wells once again were emptied, washed four times with PBST (0.1 %) for 5 min, dried, and filled with 100 µL horseradish peroxidase-conjugated anti-M13pVIII monoclonal (1:5000) in 1% BSA/PBS and incubated for one hour at RT. Finally, the wells were emptied, washed four times with PBST (0.1 %) for 5 min, dried, and filled with 50 µL TMB (development solution). The reaction was stopped after 10 min by adding 2M H_2SO_4. The absorbance changes were recorded by plate-reader at 450 nm (Synergy HTX Multi-Mode Reader by BioTek.).

2. Surface characterisation

2.1. TEM analysis

Suspensions of selected phages (10^9 pfu mL^{-1}) in PBS and corresponding peptides (1 mg mL^{-1}) in PBS were adsorbed onto carbon-coated copper grids (Sigma) for 3 min, stained with 1.5% uracyl acetate. The samples were photographed using Tecani Spirit BioTWIN microscope at 120kV, and images were processed by iTEM program.

2.2. PM-IRRS analysis (polarisation modulation infrared reflection absorption spectroscopy)

Three types of samples were prepared and analysed: (a) peptide solution in PBS (100 µg mL^{-1}), (b) CRP solution in PBS (50 µg mL^{-1}), and (c) protein-peptide complex. The complex was prepared

by mixing the protein and peptide in a 1: 1 volume ratio and incubated overnight at 4 °C. 200 μL of each sample was deposited on a glass slide covered with a 100 nm gold film vapour deposited with a pre-coated 0.5 nm chromium adhesive layer. Before the experiment, each slide was cleaned by washing it in distilled water, pure ethanol and hot distilled water. Then the respective peptide solution was left for assembling on the gold plate overnight at 4 °C. Sequentially, the CRP and the complex solutions were dropped on the clean gold plate and left for 30 min incubation at RT. After incubation, all samples were rinsed with distilled water.

The measurements were performed with the use of Thermo Nicolet 8700 spectrometer equipped with an external table-top optical mount, the MCT (mercury cadmium telluride) detector cooled with liquid nitrogen, photoelastic modulator, PEM (PM−100 Hinds Instrument, Hillsboro, USA), and synchronous sampling demodulator, SS (GWC Instruments, Madison, USA). The infrared spectra were acquired using the PEM set for the half-wave retardation at 1500 cm^{-1}, and the angle of incidence was set at 82°. Typically 500 scans were performed, and the resolution was 4 cm^{-1}. The spectrum of bare gold support has been subtracted from the spectra of the studied layers.

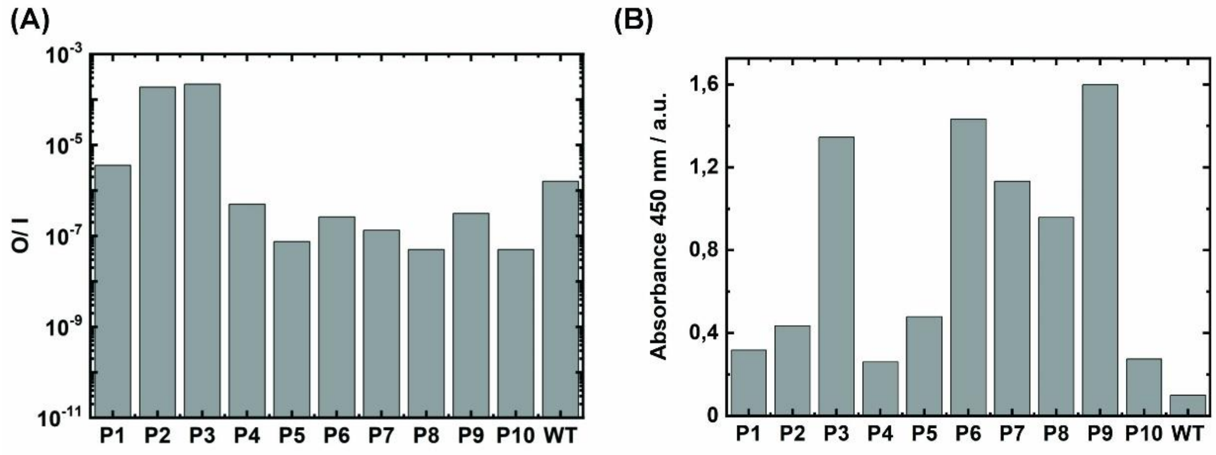

Figure S1. The affinity binding tests of the selected CRP-binding clones (P1-10) and WT. (A) The plaque test - the efficiency of CRP binding (O/I) by the selected clones and WT (B) ELISA test - analysis of the effectiveness of CRP binding by the selected clones and the WT via measurement of the absorbance.

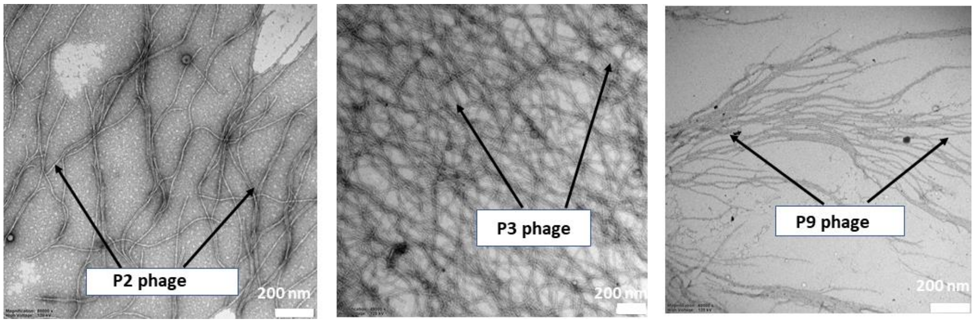

Fig. S2 TEM images of selected phages: P2 phage, P3 phage and P9 phage, scale bar: 200 nm.

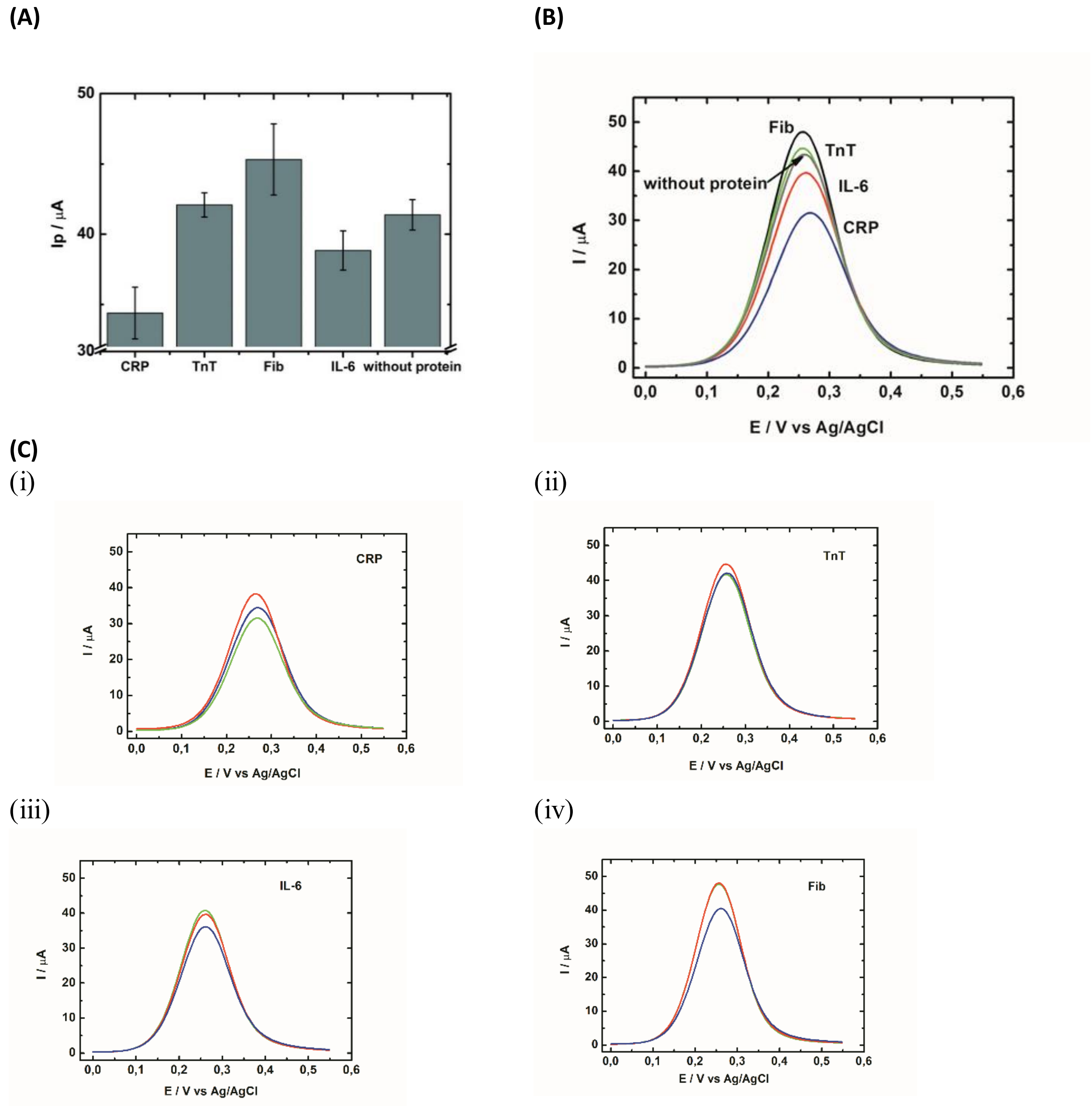

Fig. S3 (A) Selectivity test of the ITO/TESPSA/P3 in the presence of CRP (2.5 µg mL^{-1}), TnT (2.5 µg mL^{-1}), Fib (2.5 µg mL^{-1}), IL-6 (2.5 µg mL^{-1}) and without protein in 1 mM $Fc(CH_2OH)_2$/PBS. The results are average values from three experiments, with RSD represented by error bars. (B) DPV curves obtained for ITO/TESPSA/P3 electrode in the presence of 2.5 µg/mL of CRP, and 2,5 µg/mL of the interfering proteins (Fib, TnT, IL-6), and without the protein. (C) Repetitions of the measurement at three separate electrodes DPV curves measured for proteins (i CRP , (ii) TnT, (iii) IL-6, (iv) Fib).

Table S1. The binding energy of selected peptides in complex with CRP. The total surface area of the interface ($S^{int.total}$) and the hydrophobic surface area of the interface ($S^{int.hfob}$) are also specified. The binding energy and surface values for the best binding peptide and the best pose are bold.

Docking pose	Peptide	Amino acid sequence	Binding energy [kcal mol^{-1}]	$S^{int.total}$ [Å^2]	$S^{int.hfob}$ [Å^2]	$S^{int.hfob}$ / $S^{int.total}$
1	P2	GGSDPEGMQGNY[12]	-143.1	648	575	89%
2	P2	GGSDPEGMQGNY[12]	-138.7	587	526	90%
1	P3	VHWDFRQWWQPS[12]	**-174.4**	**856**	**784**	92%
2	P3	VHWDFRQWWQPS[12]	**-173.9**	769	679	88%
1	P9	SWFSDWDLELHA[12]	-162.6	728	682	94%
2	P9	SWFSDWDLELHA[12]	-160.5	808	763	94%

Table S2 Comparison of CRP biosensors based on different receptors

Receptor	Surface	Method	Range of responses	LOD	Ref.
Anti-CRP antibodies	Functionalized gold nanoparticles	CV	0,047–23,6 µg/mL	17 ng/mL	7
Anti-CRP antibodies	Dopamine-modified silica fiber	SPR	0,01-20µg/mL	n/a	8
Anti-CRP antibodies	Gold modified by bifunctional crosslinker	CV	0,01 pg/mL - 1 mg/mL	2pg/mL	9
Anti-CRP antibodies	Polyethylene terephthalate-modified ITO	EIS	21–6148 fg/mL	0.334 fg/mL	10
Anti-CRP antibodies	Bismuth citrate-modified graphite	ASV	0-100ng/mL	0,05 ng/mL	11
Anti-CRP antibodies	Polyethylenimine-modified glassy carbon	EIS<br>DPV	$1\text{-}5\cdot10^4$ ng/mL<br>$1\text{-}5\cdot10^4$ ng/mL	2,5 ng/mL<br>0,5 ng/mL	12
Anti-CRP antibodies	Polyethylene terephthalate-modified ITO	EIS	3,25–208 fg mL	0,455 fg/mL	13
Anti-CRP antibodies	Thiol-modified gold	SPR	0,006–70 mg/L	0,009 mg/L	14
Anti-CRP nanobodies	Thiol-modified gold	EIS, CV	0,25-1,5 µg/mL	0,21 µg/ml	15
CRP-Bacteriophages	Carbon nanofiber-modified glassy carbon	CV	0,04–100 µg/mL	0,04 µg/mL	2
Cysteine-labelled peptides (15 mer)	Gold	EIS	0,5–10 nmol/L	0,24nM	16
Thiolated RNA aptamers[a] /RNA aptamers[b]	Gold[a] / aminothiol-modified gold[b]	SWV	1-100 pmol/L	1 pmol/L	17
DNA aptamers	Gold	SWV	1-100 pM	1 pM	18
Biotinylated RNA aptamers	Carbon modified by streptavidin magnetic beads	SWV	0.1 – 50 mg/L	0.2 mg/L	19
Synthetic DNA aptamers	Gold modified by redox-tagged peptide	EIS	10 - 5000 pM	7.2 pM	20

biotinylated DNA/RNA aptamers	Glass modified by gold layer and linker	SPR	5-5000 fg/mL	5 fg/mL	21
RNA aptamers	Gold nanoparticles modified by functionalized silica microspheres	SWV	0.005- 125 ng/mL	0.0017 ng/mL	22
CRP-peptide (12mer)	ITO electrode modified with silicate layer (TESPSA)	DPV	1.0-100 µg/mL	0.34 µg mL^{-1}	**This work**

* - not available

References

(1) Wilson, R. K. High-Throughput Purification of M13 Templates for DNA Sequencing. *Biotechniques* **1993**, *15* (3), 414-416,418-420,422.

(2) Szot-Karpińska, K.; Kudła, P.; Szarota, A.; Narajczyk, M.; Marken, F.; Niedziółka-Jönsson, J. CRP-Binding Bacteriophage as a New Element of Layer-by-Layer Assembly Carbon Nanofiber Modified Electrodes. *Bioelectrochemistry* **2020**, *136*, 1–11. https://doi.org/10.1016/j.bioelechem.2020.107629.

(3) Piestrzyńska, M.; Dominik, M.; Kosiel, K.; Janczuk-Richter, M.; Szot-Karpińska, K.; Brzozowska, E.; Shao, L.; Niedziółka-Jonsson, J.; Bock, W. J.; Śmietana, M. Ultrasensitive Tantalum Oxide Nano-Coated Long-Period Gratings for Detection of Various Biological Targets. *Biosens. Bioelectron.* **2019**, *133*, 8–15. https://doi.org/https://doi.org/10.1016/j.bios.2019.03.006.

(4) Guillon, C.; Bigouagou, U.; Folio, C.; Jeannin, P.; Delneste, Y.; Gouet, P. A Staggered Decameric Assembly of Human C-Reactive Protein Stabilized by Zinc Ions Revealed by X-Ray Crystallography. *Protein Pept. Lett.* **2015**, *22* (3), 248–255. https://doi.org/10.2174/0929866522666141231111226.

(5) An, J.; Totrov, M.; Abagyan, R. Pocketome via Comprehensive Identification and Classification of Ligand Binding Envelopes *. *Mol. Cell. Proteomics* **2005**, *4* (6), 752–761. https://doi.org/10.1074/mcp.M400159-MCP200.

(6) Abagyan, R.; Totrov, M. Biased Probability Monte Carlo Conformational Searches and Electrostatic Calculations for Peptides and Proteins. *J. Mol. Biol.* **1994**, *235* (3), 983–1002. https://doi.org/https://doi.org/10.1006/jmbi.1994.1052.

(7) Thangamuthu, M.; Santschi, C.; Martin, O. J. F. Label-Free Electrochemical Immunoassay

for C-Reactive Protein. *Biosensors* **2018**, *8* (2). https://doi.org/10.3390/bios8020034.

(8) Wang, W.; Mai, Z.; Chen, Y.; Wang, J.; Li, L.; Su, Q.; Li, X.; Hong, X. A Label-Free Fiber Optic SPR Biosensor for Specific Detection of C-Reactive Protein. *Sci. Rep.* **2017**, *7* (1), 1–8. https://doi.org/10.1038/s41598-017-17276-3.

(9) Kim, G. W.; Zheng, S.; Kim, M. S.; Cheon, S. A.; Ko, S.; Park, T. J. Development of Specific Immobilization Method on Gold Surface and Its Application for Determining Cardiac Risk. *Biochip J.* **2014**, *8* (4), 295–302. https://doi.org/10.1007/s13206-014-8408-4.

(10) Sonuç Karaboğa, M. N.; Sezgintürk, M. K. Determination of C-Reactive Protein by PAMAM Decorated ITO Based Disposable Biosensing System: A New Immunosensor Design from an Old Molecule. *Talanta* **2018**, *186* (February), 162–168. https://doi.org/10.1016/j.talanta.2018.04.051.

(11) Kokkinos, C.; Prodromidis, M.; Economou, A.; Petrou, P.; Kakabakos, S. Disposable Integrated Bismuth Citrate-Modified Screen-Printed Immunosensor for Ultrasensitive Quantum Dot-Based Electrochemical Assay of C-Reactive Protein in Human Serum. *Anal. Chim. Acta* **2015**, *886*, 29–36. https://doi.org/10.1016/j.aca.2015.05.035.

(12) Kowalczyk, A.; Sęk, J. P.; Kasprzak, A.; Poplawska, M.; Grudzinski, I. P.; Nowicka, A. M. Occlusion Phenomenon of Redox Probe by Protein as a Way of Voltammetric Detection of Non-Electroactive C-Reactive Protein. *Biosens. Bioelectron.* **2018**, *117* (March), 232–239. https://doi.org/10.1016/j.bios.2018.06.019.

(13) Sonuç Karaboğa, M. N.; Sezgintürk, M. K. A Novel Silanization Agent Based Single Used Biosensing System: Detection of C-Reactive Protein as a Potential Alzheimer's Disease Blood Biomarker. *J. Pharm. Biomed. Anal.* **2018**, *154*, 227–235. https://doi.org/10.1016/j.jpba.2018.03.016.

(14) Aray, A.; Chiavaioli, F.; Arjmand, M.; Trono, C.; Tombelli, S.; Giannetti, A.; Cennamo, N.; Soltanolkotabi, M.; Zeni, L.; Baldini, F. SPR-Based Plastic Optical Fibre Biosensor for the Detection of C-Reactive Protein in Serum. *J. Biophotonics* **2016**, *9* (10), 1077–1084. https://doi.org/10.1002/jbio.201500315.

(15) Oloketuyi, S.; Bernedo, R.; Christmann, A.; Borkowska, J.; Cazzaniga, G.; Schuchmann, H. W.; Niedziółka-Jönsson, J.; Szot-Karpińska, K.; Kolmar, H.; de Marco, A. Native Llama Nanobody Library Panning Performed by Phage and Yeast Display Provides Binders Suitable for C-Reactive Protein Detection. *Biosensors* . 2021.

https://doi.org/10.3390/bios11120496.

(16) Piccoli, J. P.; Soares, A. C.; Oliveira, O. N.; Cilli, E. M. Nanostructured Functional Peptide Films and Their Application in C-Reactive Protein Immunosensors. *Bioelectrochemistry* **2021**, *138*, 107692. https://doi.org/10.1016/j.bioelechem.2020.107692.

(17) Jarczewska, M.; Ziółkowski, R.; Górski, Ł.; Malinowska, E. Application of RNA Aptamers as Recognition Layers for the Electrochemical Analysis of C-Reactive Protein. *Electroanalysis* **2017**, *30* (4), 658–664. https://doi.org/10.1002/elan.201700620.

(18) Jarczewska, M.; Rębiś, J.; Górski, Ł.; Malinowska, E. Development of DNA Aptamer-Based Sensor for Electrochemical Detection of C-Reactive Protein. *Talanta* **2018**, *189*, 45–54. https://doi.org/10.1016/j.talanta.2018.06.035.

(19) Centi, S.; Sanmartin, L. B.; Tombelli, S.; Palchetti, I.; Mascini, M. Detection of C Reactive Protein (CRP) in Serum by an Electrochemical Aptamer-Based Sandwich Assay. *Electroanalysis* **2009**, *21* (11), 1309–1315. https://doi.org/10.1002/elan.200804560.

(20) Piccoli, J.; Hein, R.; El-Sagheer, A. H.; Brown, T.; Cilli, E. M.; Bueno, P. R.; Davis, J. J. Redox Capacitive Assaying of C-Reactive Protein at a Peptide Supported Aptamer Interface. *Anal. Chem.* **2018**, *90* (5), 3005–3008. https://doi.org/10.1021/acs.analchem.7b05374.

(21) Vance, S. A.; Sandros, M. G. Zeptomole Detection of C-Reactive Protein in Serum by a Nanoparticle Amplified Surface Plasmon Resonance Imaging Aptasensor. *Sci. Rep.* **2014**, *4* (1), 5129. https://doi.org/10.1038/srep05129.

(22) Jia, Y.; Qin, M.; Zhang, H.; Niu, W.; Li, X.; Wang, L.; Li, X.; Bai, Y.; Cao, Y.; Feng, X. Label-Free Biosensor: A Novel Phage-Modified Light Addressable Potentiometric Sensor System for Cancer Cell Monitoring. *Biosens. Bioelectron.* **2007**, *22* (12), 3261–3266. https://doi.org/10.1016/j.bios.2007.01.018.
